# Impaired muscle strength is associated with ultrastructure damage in myositis

**DOI:** 10.1038/s41598-022-22754-4

**Published:** 2022-10-21

**Authors:** Andrea Aguilar-Vazquez, Efrain Chavarria-Avila, Mario Salazar-Paramo, Juan Armendariz-Borunda, Guillermo Toriz-González, Marcela Rodríguez-Baeza, Ana Sandoval-Rodriguez, Arisbeth Villanueva-Pérez, Marisol Godínez-Rubí, Jose-David Medina-Preciado, Ingrid Lundberg, Yesenia Lozano-Torres, Cynthia-Alejandra Gomez-Rios, Oscar Pizano-Martinez, Erika-Aurora Martinez-Garcia, Beatriz-Teresita Martin-Marquez, Sergio Duran-Barragan, Brenda-Lucia Palacios-Zárate, Arcelia Llamas-Garcia, Livier Gómez-Limón, Monica Vazquez-Del Mercado

**Affiliations:** 1 Centro Universitario de Ciencias de la Salud, Doctorado en Ciencias Biomédicas, Universidad de Guadalajara, Guadalajara, Jalisco Mexico; 2 Centro Universitario de Ciencias de la Salud, Instituto de Investigación en Reumatología y del Sistema Músculo-Esquelético (IIRSME), Universidad de Guadalajara, Guadalajara, Jalisco Mexico; 3 Departamento de Disciplinas Filosófico, Metodológicas E Instrumentales, Centro Universitario de Ciencias de la Salud, Universidad de Guadalajara, Guadalajara, Jalisco Mexico; 4 División de Medicina Interna, Servicio de Reumatología, SNP-CONACyT, Hospital Civil Dr. Juan I. Menchaca, 004086 Guadalajara, Jalisco Mexico; 5 Departamento de Fisiología, Centro Universitario de Ciencias de la Salud, Universidad de Guadalajara, Guadalajara, Jalisco Mexico; 6 Institute for Molecular Biology in Medicine and Gene Therapy, CUCS, University of Guadalajara, Guadalajara, Mexico; 7 Tecnologico de Monterrey, EMCS, Campus Guadalajara, Zapopan, Mexico; 8 Departamento de Madera, Centro Universitario de Ciencias Exactas e Ingenierías, Celulosa y Papel, Universidad de Guadalajara, Guadalajara, Jalisco Mexico; 9 Instituto Transdisciplinar de Investigaciones y Servicios, Universidad de Guadalajara, Guadalajara, Jalisco Mexico; 10 Centro de Diagnóstico e Investigación en Patología. Guadalajara, Guadalajara, Jalisco Mexico; 11 Laboratorio de Patología Diagnóstica e Inmunohistoquímica, Centro Universitario de Ciencias de la Salud, Universidad de Guadalajara, Guadalajara, Jalisco Mexico; 12 Unidad de Quemados, Hospital Civil de Guadalajara Dr. Juan I. Menchaca, Guadalajara, Mexico; 13 Departamento de Morfología, Centro Universitario de Ciencias de la Salud, Universidad de Guadalajara, Guadalajara, Jalisco Mexico; 14 Division of Rheumatology, Department of Medicine, Karolinska Institutet, Karolinska University Hospital, Solna, Stockholm, Sweden; 15 Departamento de Farmacobiología, Centro Universitario de Ciencias Exactas e Ingenierías, Universidad de Guadalajara, Guadalajara, Jalisco Mexico; 16 Centro Universitario de Ciencias de la Salud, UDG-CA 703 Inmunología y Reumatología, Universidad de Guadalajara, Guadalajara, Mexico; 17 Departamento de Clínicas Médicas, Centro Universitario de Ciencias de la Salud, Universidad de Guadalajara, Guadalajara, Mexico; 18 Departamento de Biología Molecular y Genómica, Centro Universitario de Ciencias de la Salud, Universidad de Guadalajara, Guadalajara, Mexico

**Keywords:** Autoimmunity, Chemokines, Cytokines, Rheumatology

## Abstract

The muscle fiber ultrastructure in Idiopathic Inflammatory Myopathies (IIM) has been scarcely explored, especially in Inclusion Body Myositis. The aim of this study was to implement the Scanning Electron Microscopy (SEM) in a small cohort of IIM patients, together with the characterization of immunological profile for a better understanding of the pathophysiology. For immunological profile characterization, we identified the presence of autoantibodies (Ro-52, OJ, EJ, PL7, PL12, SRP, Jo-1, PMScl75, PMScl100, Ku, SAE1, NXP2, MDA5, TIF1γ, Mi-2α, Mi-2β) and quantified cytokines (IL-1β, IFN-α2, IFN-γ, TNF-α, IL-6, IL-10, IL-12p70, IL-17A, IL-18, IL-23, IL-33) and chemokines (CCL2, CXCL8). The histological analysis was made by hematoxylin–eosin staining while the muscle fiber ultrastructure was characterized by SEM. We observed changes in the morphology and structure of the muscle fiber according to muscle strength and muscle enzymes. We were able to find and describe muscle fiber ultrastructure with marked irregularities, porosities, disruption in the linearity and integrity of the fascicle, more evident in patients with increased serum levels of muscle enzymes and diminished muscle strength. Despite the scarce reports about the use of SEM as a tool in all clinical phenotypes of IIM, our work provides an excellent opportunity to discuss and reframe the clinical usefulness of SEM in the diagnostic approach of IIM.

## Introduction

Idiopathic Inflammatory Myopathies (IIM) also known as myositis, are muscle autoimmune diseases with different clinical, demographical, histological, and immunopathological features that allow us to classify them into subgroups: dermatomyositis, amyopathic dermatomyositis (ADM), juvenile dermatomyositis (JDM), polymyositis (PM), inclusion body myositis (IBM), immune-mediated necrotizing myopathy (IMNM) and juvenile myositis (JM)^1^.

Distinct classification criteria have been proposed by experts in this field through the years, which allows the clinical diagnosis, notwithstanding still is scarce the information available regarding the pathogenesis of the disease. The diagnostic tools used for IIM include electromyography, magnetic resonance imaging, serum levels of muscle enzymes, presence of autoantibodies and muscle histological characterization; however, they may not be specific enough for some IIM phenotypes according to classification criteria^2^.

There are still many fields to explore deeply in IIM research, for example, the immunological profile of IIM patients, including the clinical relevance of the myositis specific antibodies (MSA) beyond cancer risk development or anti-synthetase syndrome (ASS) and myositis associated antibodies (MAA), as well as the participation of cytokines and chemokines. Notwithstanding, the histological analysis of the muscle is commonly performed by hematoxylin–eosin (HE) staining, however, the information about the description of muscle fiber ultrastructure is scarce and limited to IBM. There are only a few descriptive reports regarding muscle fiber in other pathologies or conditions^3–6^. The Scanning Electron Microscopy (SEM) has not been fully explored, since the lack of availability in clinical centers and the expertise needed for its interpretation represent major limitations.

Some reports have implemented transmission electron microscopy (TEM), which is useful particularly for IBM patients due to their classic inclusion bodies structures^7^; likewise, a few reports have included muscle analysis of DM and PM patients^8^. However, neither of these studies described rigorously the muscle fiber ultrastructure of IIM patients using SEM, therefore our study represents the first report in this context.

The use of SEM will allow us, with the highest resolution, to appropriately characterize the muscle fiber ultrastructure, and along with cytokines, chemokines and the presence of autoantibodies, might provide us a better understanding of the pathological features of IIM. Therefore, our study aim was to characterize the muscle fiber ultrastructure and to identify the possible association with the immunological profile and clinical features of the IIM clinical phenotypes.

## Results

Our study group was conformed of eight women (66.7%) and four men (33.3%) classified with IIM according to EULAR/ACR 2017 criteria. Regarding extra muscle manifestations, we found that arthralgias and interstitial lung disease (ILD) were the most prevalent. Other demographical, clinical, and serological features are shown on Table 1.

**Table 1:** Clinical and serological features of patients classified with Idiopathic Inflammatory Myopathies in our study group.

Clinical or serological feature	Men (n = 4)	Women (n = 8)	Both (n = 12)	P*
Age (years,$\overline{\mathrm{x }}\pm \mathrm{S }.\mathrm{D}.$)	39.5 ± 18.19	44.7 ± 14.28	43.0 ± 15.06	0.461^#^
MMT8 (score, $\overline{\mathrm{x }}\pm \mathrm{S }.\mathrm{D}.$)	137.5 ± 21.19	144.6 ± 5.50	142.2 ± 12.41	0.972^#^
CPK (U/L, $\overline{\mathrm{x }}\pm \mathrm{S }.\mathrm{D}.$)	232.2 ± 219.29	346.87 ± 695.07	308.7 ± 568.98	0.570^#^
Aldolase (U/L, $\overline{\mathrm{x }}\pm \mathrm{S }.\mathrm{D}.$)	5.07 ± 3.30	3.5 ± 2.06	4.2 ± 2.54	0.629^#^
AST (U/L, $\overline{\mathrm{x }}\pm \mathrm{S }.\mathrm{D}.$)	39.2 ± 21.73	27.5 ± 12.90	31.4 ± 16.38	0.715^#^
ALT (U/L, $\overline{\mathrm{x }}\pm \mathrm{S }.\mathrm{D}.$)	62.4 ± 46.67	22.3 ± 9.47	35.6 ± 32.28	0.117^#^
LDH (U/L, $\overline{\mathrm{x }}\pm \mathrm{S }.\mathrm{D}.$)	173.3 ± 30.01	253.0 ± 96.79	229.1 ± 89.03	0.383^#^
CRP (mg/L, $\overline{\mathrm{x }}\pm \mathrm{S }.\mathrm{D}.$)	6.3 ± 7.20	10.5 ± 16.85	8.9 ± 13.80	0.927^#^
ESR (mm/h, $\overline{\mathrm{x }}\pm \mathrm{S }.\mathrm{D}.$)	12.2 ± 15.35	30.0 ± 15.82	23.5 ± 17.35	0.048^#^
**Personal history**				
Diabetes n(%)	–	–	–	–
Hypertension n(%)	1 (25.0)	–	1 (8.3)	–
Alcohol n(%)	2 (50.0)	–	2 (16.7)	–
Smoke n(%)	3 (75.0)	–	3 (25.0)	–
Drugs n(%)	2 (50.0)	–	2 (16.7)	–
**Extramuscular manifestations**				
Arthritis n (%)	1 (25.0)	2 (25.0)	3 (25.0)	–
Arthralgia n (%)	–	4 (50.0)	4 (33.3)	–
Joint contracture n (%)	–	–	–	–
Mechanic's hands n (%)	–	–	–	–
Raynaud's phenomenon n (%)	–	2 (25.0)	2 (16.7)	–
Heliotrope n (%)	1 (25.0)	1 (12.5)	2 (16.7)	–
Gottron's sign n (%)	1 (25.0)	1 (12.5)	2 (16.7)	–
Shawl sign n (%)	–	1 (12.5)	1 (8.3)	–
V sign n (%)	1 (25.0)	2 (25.0)	3 (25.0)	–
Periungual erythema n (%)	1 (25.0)	–	1 (8.3)	–
Ulceration n (%)	–	–	–	–
Gastrointestinal perforation n (%)	–	–	–	–
Abdominal pain n (%)	–	–	–	–
Dysphagia n (%)	1 (25.0)	1 (12.5)	2 (16.7)	–
Calcinosis n (%)	–	1 (12.5)	1 (8.3)	–
Interstitial lung disease n (%)	1 (25.0)	3 (37.5)	4 (33.3)	–
Pulmonary fibrosis n (%)	2 (50.0)	2 (25.0)	4 (33.3)	–
Pulmonary hypertension n (%)	–	1 (12.5)	1 (8.3)	–
Vasculitis n (%)	–	–	–	–
Arrhythmias n (%)	–	–	–	–
Cardiomyopathy n (%)	–	–	–	–
Myocardial infarction n (%)	–	–	–	–
Associated cancer n (%)	–	1 (25.0)	1 (8.3)	–
**Treatment**				
Methotrexate n (%)	2 (50.0)	7 (87.5)	9 (75.0)	0.300^&^
Prednisone n (%)	3 (75.0)	5 (62.5)	8 (66.7)	> 0.999^&^
Hydroxychloroquine n (%)	1 (25.0)	3 (37.5)	4 (33.3)	–
Mycophenolate n (%)	1 (25.0)	2 (25.0)	3 (25.0)	–
Azathioprine n (%)	–	1 (12.5)	1 (8.3)	–
**Supplement**				
Folic acid n (%)	2 (50.0)	6 (75.0)	8 (66.7)	> 0.999^&^
Vitamin D n (%)	3 (75.0)	3 (37.5)	6 (50.0)	0.200^&^

*IIM* idiopathic inflammatory myopathies; $\overline{x}$ mean; *S.D.* standard deviation; *MMT8* Manual Muscle Testing 8; *CPK* creatine phosphokinase; *AST* aspartate aminotransferase; *ALT* alanine aminotransferase; *LDH* lactate dehydrogenase; *CRP* C-reactive protein; *ESR* erythrocyte sedimentation rate.

*Only the characteristics of men and women were included in the statistical analysis.

^#^Mann–Whitney U with Fisher's Exact test.^&^Fisher's exact for contingency tables test.

### There is a negative correlation between muscle strength and muscle enzymes

**Figure 1:**
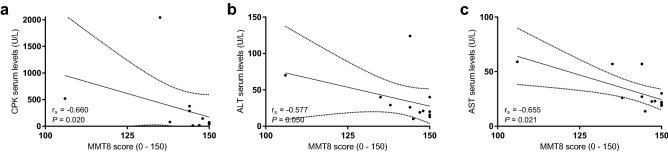
Correlation between muscle damage and muscle weakness markers. (**a**) serum levels of CPK and MMT score; (**b**) ALT serum levels and MMT8 score; (**c**) serum levels of AST and MMT8 score. MMT8 Manual Muscle Testing 8; CPK creatine phosphokinase; ALT alanine aminotransferase; AST aspartate aminotransferase.

We determined the serum levels of muscle enzymes (CPK, aldolase, AST, ALT, LDH) and MMT8 score (0–150). We found an inverse correlation between the MMT8 score and CPK (r_s_ = − 0.660, *P* = 0.020), ALT (r_s_ = − 0.577, *P* = 0.050) and AST (r_s_ = − 0.655, *P* = 0.021), meaning that patients with higher levels of muscle enzymes had lower muscle strength (Fig. 1 panel a, b, and c).

### Findings in muscle fiber ultrastructure became more evident in patients with diminished muscle strength

**Figure 2:**
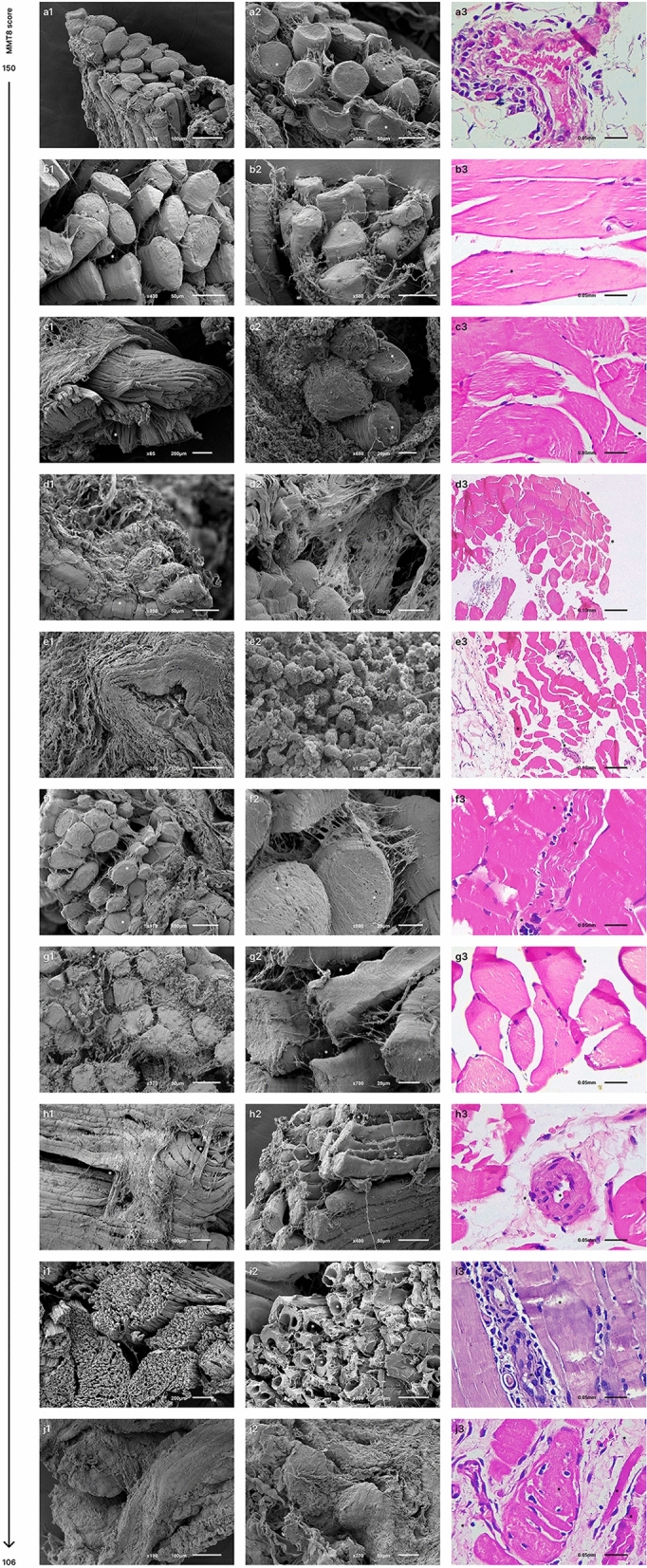
Muscle fiber ultrastructure observed by scanning electron microscopy and tissue analysis performed by hematoxylin–eosin stain according to MMT8 score. (**a1**, **a2**) Small porosities and irregularities. (**a3**)**.** Mild perifascicular atrophy, scarce perivascular inflammatory infiltrate, and necrotic fiber clusters. (**b1**, **b2**). Muscle fibers were dispersed, non-linear and they had distinct morphology with abundant cell infiltrate. Small porosities and marked irregularities. (**b3**). There were no suggestive morphological alterations of muscle pathology. (**c1**, **c2**)**.** Marked irregularities and non-linear muscle fibers altering the morphology of the fascicle, as well as abundant cell infiltrate. (**c3**)**.** Edema and bleeding however there were no evidence of muscle pathology. (**d1**, **d2**)**.** We did not observe the characteristic structure of the muscle fascicle. There were abundant irregularities in structure, size, arrangement, and linearity, and abundant fibrin deposits. (**d3**)**.** Scarce perivascular inflammatory infiltrate in perifascicular vessels with mild local atrophy of perifascicular fibers. (**e1**, **e2**)**.** Muscle myofibers distribution was not lineal, they were dispersed with multiple surface irregularities and abundant leukocyte infiltrate. (**e3**)**.** Muscle tissue analysis denoted inflammation foci (perifascicular and endomysial) with scarce endomysial inflammatory infiltrate. Muscle fiber atrophy with perifascicular predominance. (**f1**, **f2**)**.** Irregularities in sarcolemma and multiple surface porosities. (**f3**)**.** Mild endomysial inflammation with an increment of connective tissue and discrete muscle fiber size variation. Scarce degenerated myofibers. (**g1**, **g2**)**.** Irregularities in sarcolemma linearity and a considerable number of cellular clusters. (**g3**)**.** There were no suggestive morphological alterations of muscle pathology. (**h1**, **h2**)**.** Sarcolemma irregularities, disruption muscle fibers linearity and leukocyte infiltrate. (**h3**)**.** Scarce perivascular inflammatory infiltrate and endothelial hyperplasia in small-caliber vessels. (**i1**, **i2**)**.** Muscle fiber destruction with multiple perforations. (**i3**)**.** Perivascular inflammatory infiltrate in perifascicular and endomysial vessels, scarce chronic endomysial inflammatory, infiltrate, and presence of basophilic fibers in regeneration without atrophy evidence. (**j1**, **j2**)**.** Multiple surface myofiber irregularities. (**j3**)**.** Diffuse and multifocal atrophy. Muscle fibers exhibited marked size and shape differences. Atrophy was not limited to the perifascicular zone. Mild chronic endomysial inflammatory infiltrate, some muscle fibers had hydropic tumefaction evidence without perivascular inflammation. Basophilic muscle fibers with regenerative aspects.

The muscle tissue of twelve patients was analyzed by optical microscopy stained with HE and ten samples were also analyzed by SEM. Most representative biopsy pictures of our patients are shown according to MMT8 score in Fig. 2. In this regard, it is important to denote that we observed higher levels of muscle enzymes and more evident findings (porosities, sarcolemma irregularities, perforations, and even loss of muscle morphology) in the muscle fiber ultrastructure of patients with lower muscle strength. We did not observe an association between ultrastructural alterations and the presence of autoantibodies, nor myositis phenotype. Clinical features of each patient according to the MMT8 score are shown on Table 2.

**Table 2:** Clinical features according to MMT8 score decreasing order.

Patient	Age	Gender	Presence of MSA/MAA	Extra muscular manifestations	Myositis classification	MYOACT score	MDI score*	MMT8 score (0–150)	Serum levels of muscle enzymes
A	22	Male	Anti-MDA5	Heliotrope, Gottron’s sign, V sign, periungual erythema	ADM	0	0	150	**Normal:** CPK = 50.0 U/L; AST = 18.9 U/L; ALT = 15.7 U/L; LDH = 206.0 U/L; aldolase = 1.7 U/L
B	34	Male	Seronegative	Arthritis	IBM	0	0	150	**Normal:** CPK = 73.0 U/L; AST = 22.0 U/L; ALT = 40.0 U/L; LDH = 147.0 U/L; aldolase = 8.3 U/L
C	39	Female	Seronegative	Arthralgia, heliotrope, Gottron’s sign, shawl’s sign, calcinosis, ILD	DM	0	0.03	148	**Normal:** CPK = 145.0 U/L; AST = 23.0 U/L; ALT = 21.0 U/L; LDH = 160.0 U/L
D	51	Female	Anti-Ro-52	Arthritis, arthralgia, V sign	OM	0	0	148	**Normal:** CPK = 18.0 U/L: AST = 22.7 U/L; ALT = 19.1 U/L; LDH = 192.0 U/L
E	36	Female	Seronegative	Arthralgia, V sign, Raynaud’s phenomenon	OM	0.03	0	145	**Normal:** CPK = 13.0 U/L; AST = 13.7 U/L; ALT = 10.1 U/L; LDH = 225.0 U/L
F	37	Male	Seronegative	ILD, pulmonary fibrosis	DM	0.05	0.05	144	**Elevated:** CPK = 286.0 U/L; AST = 57.0 U/L; ALT = 124.0 U/L; LDH = 167.0 U/L; aldolase = 5.2 U/L
G	49	Female	Seronegative	Arthritis, arthralgia, Raynaud’s phenomenon	OM	0	0	144	**Elevated:** CPK = 376.0 U/L; AST = 27.0 U/L; ALT = 26.0 U/L
H	48	Female	Seronegative	No extra muscular manifestations	PM	0.03	0.03	138	**Normal:** CPK = 79.0 U/L; AST = 26.0 U/L; ALT = 29.0 U/L; LDH = 145.0 U/L; aldolase = 1.4 U/L
I	45	Female	Anti-EJ Anti-Ro-52	Dysphagia, ILD, pulmonary fibrosis, pulmonary hypertension	PM + ASS	0.28	0.20	135	**Elevated:** CPK = 2042.0 U/L; AST = 57.0 U/L; ALT = 40.0 U/L; LDH = 391.0 U/L; aldolase = 5.6
J	65	Male	Seronegative	Dysphagia, pulmonary fibrosis	PM	0.33	0.23	106	**Elevated:** CPK = 520.0 U/L; AST = 59.0 U/L; ALT = 70.0 U/L
–	70	Female	Anti-EJ Anti-Ro-52	ILD, pulmonary fibrosis, cancer	PM + ASS	0.02	0	150	**Elevated:** CPK = 39.0 U/L; AST = 20.3 U/L; ALT = 20.1 U/L; LDH = 309.0 U/L; aldolase = 2.1 U/L
–	20	Female	Anti-TIF-1γ	No extra muscular manifestations	DM	0	0	150	**Elevated:** CPK = 63.0 U/L; AST = 30.1 U/L; ALT = 12.8 U/L; LDH = 349.0 U/L; aldolase = 4.9 U/L

*MSA* Myositis Specific Autoantibodies; *MAA* Myositis Associated Autoantibodies; *MDA5* Melanoma Differentiation Associated-protein 5; *TIFγ1* Transcription Intermediary Factor 1γ; *ILD* Interstitial Lung Disease; *ADM* amyopathic dermatomyositis; *IBM* Inclusion Body Myositis; *DM* dermatomyositis; *OM* Overlap Myositis; *PM* polymyositis; *ASS* anti-synthetase syndrome; *MYOACT* Myositis Disease Activity Assessment Visual Analogue Scales; *MDI* Myositis Damage Index; *MMT8* Manual Muscle Testing 8; *CPK* creatin phosphokinase; *AST* aspartate aminotransferase; *ALT* alanine aminotransferase; *LDH* Lactate Dehydrogenase.

*MDI extent of damage score.

### Cytokine and chemokine profile: IL-6 serum levels are higher in myositis patients meanwhile IFN-γ and CCL2 are higher when positive MSA/MAA

Regarding immunological profile characterization, we detected the serum presence of MSA/MAA and quantified serum levels of cytokines and chemokines. We found five seropositive patients for MSA or MAA, which represented 41.7% of seropositivity in our study group, the clinical features of seropositive patients are also shown on Table 2.

**Figure 3:**
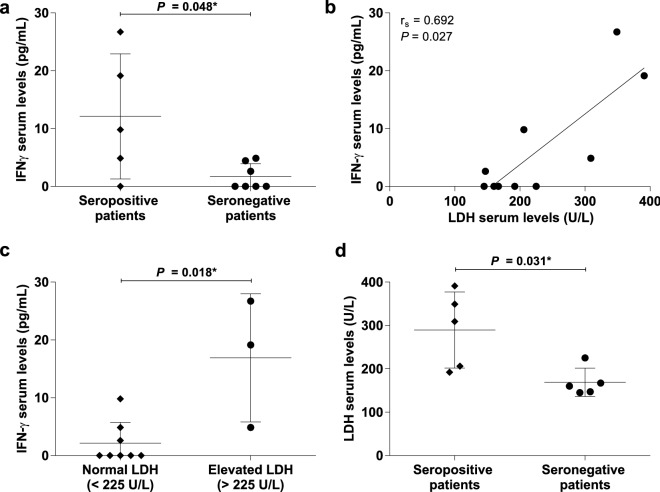
Association between LDH enzyme as muscle damage marker and immunological profile (autoantibodies and cytokines) of IIM patients. (**a**) Higher serum levels of IFN-γ in MSA/MAA seropositive patients; (**b**) higher serum levels of LDH in MSA/MAA seropositive patients; (**c**) correlation between IFN-γ and LDH serum levels, and; (**d**) higher serum levels of IFN-γ in patients with elevated levels of LDH. LDH Lactate Dehydrogenase; IIM Idiopathic Inflammatory Myopathies; IFN interferon; MSA Myositis Specific Autoantibodies; MAA Myositis Associated Autoantibodies.*Mann-Whitney U test with Fisher’s Exact test.

Regarding the characterization of cytokines and chemokines profile, we included a control group conformed by twelve clinically healthy subjects (age- and gender-matched) to compare the serum levels of these molecules in IIM patients. We found higher serum levels of IL-6 in IIM patients (5.0 ± 4. 33 *vs.* 11.3 ± 7.69 pg/mL, *P* = 0.028) (Table 3). Subsequently, we compared the cytokine and chemokine serum levels according to seropositivity for any MSA or MAA *vs.* seronegative patients (Supplementary Table 1). We found higher levels in seropositive patients of CCL2 (294.8 ± 85.11 *vs.* 116.3 ± 66.41 pg/mL, *P* = 0.005) and IFN-γ (12.1 ± 10.80 *vs.* 1.7 ± 2.23 pg/mL *P* = 0.048) (Fig. 3 panel a).

**Table 3:** Cytokine and chemokine serum levels in IIM patients and control group.

Serum levels (pg/mL)	IIM patients (n = 12)	Detection rate n (%)	Control group (n = 12)	Detection raten (%)	P*^#^	*P*^$&^
IL-1β ($$\overline{{\text{x }}} \pm {\text{S}}.{\text{D}}.$$)	3.1 ± 5.66	5 (41.6)	3.6 ± 6.57	5 (41.6)	0.892	> 0.999
IFN-α2 ($$\overline{{\text{x }}} \pm {\text{S}}.{\text{D}}.$$)	2.0 ± 1.41	12 (100.0)	3.3 ± 3.84	12 (100.0)	0.600	–
IFN-γ ($$\overline{{\text{x }}} \pm {\text{S}}.{\text{D}}.$$)	6.0 ± 8.59	7 (58.3)	5.2 ± 8.44	5 (41.6)	0.615	0.684
TNF-α ($$\overline{{\text{x }}} \pm {\text{S}}.{\text{D}}.$$)	0.0	0	3.8 ± 8.97	2 (16.6)	–	0.478
IL-6 ($$\overline{{\text{x }}} \pm {\text{S}}.{\text{D}}.$$)	11.3 ± 7.69	12 (100.0)	5.0 ± 4.33	8 (66.7)	0.028	0.317
IL-10 ($$\overline{{\text{x }}} \pm {\text{S}}.{\text{D}}.$$)	1.2 ± 1.27	11 (91.6)	1.7 ± 3.73	6 (50.0)	0.187	0.069
IL-12p70 ($$\overline{{\text{x }}} \pm {\text{S}}.{\text{D}}.$$)	0.0	0	0.6 ± 1.05	3 (25.0)	0.217	0.217
IL-17A ($$\overline{{\text{x }}} \pm {\text{S}}.{\text{D}}.$$)	0.2 ± 0.35	3 (25.0)	1.2 ± 2.28	6 (50.0)	0.200	0.400
IL-18 ($$\overline{{\text{x }}} \pm {\text{S}}.{\text{D}}.$$)	383.9 ± 665.9	11 (91.6)	144.6 ± 75.7	12 (100.0)	0.291	> 0.999
IL-23 ($$\overline{{\text{x }}} \pm {\text{S}}.{\text{D}}.$$)	26.4 ± 80.65	12 (100.0)	2.6 ± 3.08	11 (91.6)	0.214	> 0.999
IL-33 ($$\overline{{\text{x }}} \pm {\text{S}}.{\text{D}}.$$)	3.8 ± 7.41	3 (25.0)	4.6 ± 10.73	2 (16.6)	> 0.999	> 0.999
CCL2 ($$\overline{{\text{x }}} \pm {\text{S}}.{\text{D}}.$$)	190.6 ± 116.11	12 (100.0)	116.1 ± 44.20	12 (100.0)	0.128	–
CXCL8 ($$\overline{{\text{x }}} \pm {\text{S}}.{\text{D}}.$$)	3.1 ± 4.12	8 (66.7)	5.9 ± 11.87	9 (75.0)	0.919	> 0.999

*MSA* Myositis Specific Autoantibodies; *MAA* Myositis Associated Autoantibodies; *IIM* Idiopathic Inflammatory Myopathies; $\overline{x }$ mean; *S.D.* standard deviation; *pg* picogram; *mL* milliliter; *IL* interleukin; *IFN* interferon; *TNF* Necrosis Tumor Factor; *CCL2* chemokine (C–C motif) ligand 2; *CXCL8* chemokine (C-X-C motif) ligand 8.

*We compared cytokine and chemokine serum levels between IIM patients and control group.

^#^Mann–Whitney U test with Fisher’s exact test.

^$^We compared the detection rate of cytokines and chemokines between IIM patients and control group. ^&^Fisher’s exact test.

### Correlation of cytokines and chemokines with muscle damage markers

Since we observed marked alterations in muscle fiber ultrastructure in patients with higher muscle enzymes and lower muscle strength, we analyzed if there are any associations between these findings with the immunological profile. We initially looked for a possible correlation between muscle enzymes or MMT8 score with serum levels of cytokines or chemokines. We did not find any correlation regarding cytokines or chemokines with CPK, AST, ALT nor MMT8 score, however, we observed a correlation between serum levels of IFN-γ and LDH (r_s_ = 0.692, *P* = 0.027) (Fig. 3 panel b) as well as a negative correlation between CXCL8 and aldolase (r_s_ = − 0.929, *P* = 0.003).

Subsequently, we analyzed cytokines and chemokines serum levels according to normal or elevated muscle enzymes. We found higher levels of IFN-γ (16.9 ± 11.09 pg/mL vs. 2.2 ± 3.6 pg/mL, *P* = 0.018) (Fig. 3 panel c) in patients with elevated levels of LDH.

Regarding the muscle enzyme serum levels according to MSA/MAA presence, we found higher serum levels of LDH (289.4 ± 87.61 vs. 168.8 ± 32.71 U/L, *P* = 0.031) in seropositive patients (Fig. 3 panel d). We did not find difference in the scale of the MMT8 score according to seropositivity (*P* = 0.216). On the other hand, we did not find difference in the frequency distribution of MSA/MAA seropositivity according to normal or elevated levels of muscle enzymes.

All these findings give us a picture indicating that MSA/MAA seropositive patients have higher levels of the cytokine IFN-γ as well as the muscle enzyme LDH (Fig. 3), which is consistent with progressive alterations in muscle fiber ultrastructure according to the MMT8 score.

### Histopathological findings in muscle tissue are in concordance with the muscle damage and MMT8 score of IIM patients

We additionally looked for any association between a specific finding in muscle tissue and the clinical and immunological profile of our patients.

According to HE results, we observed that the most common histopathological features were perivascular inflammatory infiltrate (50%), perifascicular atrophy (41.7%), endomysial inflammatory infiltrate (33.3%) and the presence of basophilic fibers (25%).

We observed that patients with perivascular inflammatory infiltrate had higher erythrocyte sedimentation rate (ESR) (37.8 ± 9.73 mm/h vs. 11.7 ± 12.40 mm/h, *P* = 0.015). Regarding patients with endomysial inflammatory infiltrate, we found higher levels of IL-18 (841.5 ± 1084.19 vs. 155.2 ± 117.03 pg/mL, *P* = 0.048) and lower score of MMT8 (132.5 ± 18.23 vs. 147.1 ± 4.26, *P* = 0.034). Finally, we observed higher serum levels of CPK (949.3 ± 953.48 U/L vs. 95.1 ± 112.38 U/L, *P* = 0.018), AST (57.7 ± 1.15 U/L vs. 22.6 ± 4.82, *P* = 0.009) and ALT (78.0 ± 42.6 U/L vs. 21.5 ± 9.12 U/L, *P* = 0.014) as well as lower score of MMT8 (128. 3 ± 19.86 vs. 146.9 ± 4.04, *P* = 0.032) in patients with basophilic fibers.

Regarding SEM observations, muscle fibers were cylindrical, with a diameter of 50 μm approximately, parallel to each other and they were surrounded by collagen fibers forming the endomysium. The most common finding was myofiber surface irregularities (90%), however, since this feature is present in almost all of our patients, it is considered characteristic of muscle fiber ultrastructure independently of the clinical phenotype.

Other findings included altered muscle morphology (60%), non-linear muscle fibers (60%), cellular infiltrate (50%), and myofiber surface porosities (30%). In this regard, in patients with altered muscle morphology, we observed non detectable levels of IL-1β (0.0 pg/mL vs. 4.8 ± 4.23 pg/mL, *P* = 0.005) as well as lower levels of CXCL8 (0.8 ± 1.20 pg/mL vs. 5.6 ± 5.73 pg/mL, *P* = 0.024).

### The immunological profile of IIM patients is also related to immunosuppressive treatment

We also decided to explore if there is an association between the immunological profile of IIM patients and clinical characteristics including immunosuppressive treatment.

The patients included in this study are under conventional synthetic disease-modifying drugs including methotrexate, prednisone, hydroxychloroquine, mycophenolate, and azathioprine. We looked for differences in cytokine and chemokine serum levels as well as the frequency of seropositivity. We could not compare methotrexate because almost all our patients were under this treatment (Supplementary Table 2). We found lower levels of chemokine CXCL8 in prednisone- treated patients (1.6 ± 1.50 pg/mL vs. 8.3 ± 5.59 pg/mL, *P* = 0.018), as well as a tendency of diminished serum levels of IL-23 in mycophenolate mofetil-treated patients (1.2 ± 0.48 pg/mL vs. 39.1 ± 98.33 pg/mL, *P* = 0.085); moreover, although no significative, serum levels of IL-1β and IL-17A were not detectable in patients under mycophenolate treatment. We did not observe differences concerning hydroxychloroquine. Regarding MSA/MAA presence, we did not observe a difference in the frequency distribution of autoantibody seropositivity according to immunosuppressive treatment.

## Discussion

To our knowledge, this study is the first one to describe the muscle fiber ultrastructure of IIM patients in clinical phenotypes ADM, DM, JDM, PM plus anti-synthetase syndrome (ASS) and overlap myositis (OM) by SEM. Since the unexplored clinical use of SEM in these patients and the lack of classification criteria including SEM findings, we decided to categorize and describe the muscle fiber ultrastructure according to muscle damage and muscle weakness markers represented by muscle enzymes and MMT8 score, respectively. To complement the obtained information on muscle fiber ultrastructure, we also characterized the immunological profile of IIM patients aiming to get a better understanding of their pathophysiology.

Serum levels of muscle enzymes are considered a biomarker for myositis classification considered in EULAR/ACR 2017 criteria. Is important to remind that serum levels of muscle enzymes might reflect the muscle damage or the lack of physical training in some IIM patients^9^, meanwhile the MMT8 score is a validated tool for muscle weakness evaluation in myositis patients^10^, therefore both parameters are considered adequate markers for muscle damage and muscle weakness.

According to muscle fiber ultrastructure description, we presented the SEM images in decreasing order of the MMT8 score (Fig. 2). Only two of ten patients had a MMT8 maximum score (150), the muscle tissue of these patients included muscle fibers with lineal morphology, similar size and were disposed of in fascicle conformation. However, while muscle strength decreased, we observed more irregularities, porosities, abundant leukocyte infiltration as well as disruption of linearity and fascicle conformation, even in the patients with the lowest MMT8 score. We observed muscle fiber destruction with multiple perforations and difficulty to identify muscle fiber and muscle fascicle structures. Likewise, muscle enzymes elevation became evident as muscle strength decreased, this is in concordance with the observed correlation between serum levels of muscle enzymes and the MMT8 score (Fig. 1).

An important aspect to consider regarding the muscle fiber description of our patients is the fact of the observed discordances between optical and electronic microscopy; according to HE staining, we did not observe any finding suggestive of muscle pathology in three of our patients, meanwhile, we found alterations in the ultrastructure of muscle fiber of the same patients. This situation denotes the usefulness of SEM as a tool with higher resolution to get a better opportunity to observe histological changes and a better comprehension of the muscle tissue alterations of IIM patients.

Another important fact regarding muscle histological examination of IIM patients is the discordance between clinical and histopathological diagnosis that has been previous reported, indicating up to 55% of change in diagnosis after muscle biopsy in IIM patients. Muscle biopsy examination has also a great utility in deep characterization of IIM patients^11^. In our study, the clinical phenotype was not concordant in 30% with muscle biopsy results; this is one of the reasons why the decision of muscle biopsy is debatable nowadays, due to the lack of expertise in its interpretation. These findings highlight the importance of muscle biopsy for an accurate diagnosis and classification of IIM patients.

In addition to muscle fiber description, we also aimed to characterize the immunological profile of our study group by serum detection of MSA and MAA as well as cytokines and chemokines quantification. We found a seropositivity of 41.7% of MSA or MAA in concordance with the phenotypes previously described as the case of the presence of anti-MDA5 in ADM phenotype or ILD development in anti-EJ seropositive patients^12,13^.

Regarding cytokines, it is important to denote that not all cytokines or chemokines were detected because their serum levels were lower than the minimum considered in the detection range, we considered them as zero for comparisons and reported the detection rate in Table 3. In this context, cytokines such as IFN-α2, IL-6, IL-23, and chemokine CCL2 had a detection rate of 100%, meanwhile IL-12p70 and TNF-α were not detected in the serum of IIM patients and the cytokine IL-17 was detected in fewer patients probably due to immunosuppressive therapy.

We found higher levels of IL-6 in IIM patients respecting to control group. This cytokine is one of the main mediators of pro-inflammatory responses, mainly secreted by macrophages, fibroblasts and endothelial cells^14^; IL-6 promotes vascular endothelial activation by an increase of E-selectin, intercellular adhesion molecule 1 (ICAM-1) and vascular cellular adhesion molecule 1 (VCAM-1) expression which is important for leukocyte migration to muscle tissue^15^, otherwise, IL-6 elevated levels have been reported in other rheumatic diseases including rheumatoid arthritis, Sjögren’s syndrome, and Crohn’s disease^16–18^. The role of IL-6 in IIM has been previously reported as the case of the model of myosin-induced experimental myositis where mice developed myopathy, but IL-6 deficient mice did not show clinical nor histological signs of muscle damage^19^. Additionally, elevated serum levels of IL-6 in IIM patients have been also reported and proposed as an activity disease biomarker in DMJ^20^. Considering this background as well as our results, we could consider IL-6 as a one of the main mediators of tissue destruction in IIM immunopathology.

Otherwise, IFNγ and IL-17A, which are related cytokines to IIM pathogenesis because of their participation in MHC-I overexpression and autoimmunity development^21,22^, did not show difference. This behavior might be explained by the clinical remission of the IIM, the immunosuppressive treatment or the low concentrations of cytokines and chemokines in peripheral blood, however, it would be important to determine the expression of these molecules directly in muscle tissue.

Afterwards, according to MSA/MAA seropositivity, we observed only a tendency of IL-6 higher levels in seropositive patients. On the other hand, we found higher levels of IFN-γ and CCL2 in seropositive patients. To our knowledge, this is the first report comparing cytokine and chemokine serum levels according MSA/MAA seropositivity, however, due to small sample size, we could not carry out the analysis for each autoantibody. We propose the comparative of cytokine and chemokine serum levels according to each MSA and MAA as a perspective for a better understanding of the immunological profile of IIM patients.

The implication of IFNs in IIM is well known and has been widely reported since the first time in 1986^23^. This cytokine is mainly produced by monocytes, macrophages, and natural killer^24^ cells after pattern recognition receptors (PRR) activation, but IFN-γ is also synthesized by TCD4^+^ lymphocytes (Th1 profile) and TCD8^+^ lymphocytes^25^. It has been demonstrated that the IFN-γ gene is overexpressed in myofibers surrounded by TCD8^+^ lymphocytes^26^. Otherwise, it is highly recognized that this cytokine induces the MHC-I and MHC-II overexpression in human myoblasts^27,28^, which is associated with IIM immunopathology because it facilitates myofibers recognition by TCD8^+^ lymphocytes^15^, and triggers endoplasmic reticulum stress^29^.

On the other hand, it has been reported that the chemokine CCL2, mainly produced by monocytes and macrophages, participates in IIM pathogenesis by the stimulation of leukocyte migration to the muscle tissue^30^. In addition, the inflammatory infiltrates which surround the blood vessels of muscle tissue are mainly composed of lymphocytes and macrophages, which are target cells of CCL2^15^. It has been shown distinct evidence of CCL2 implication in myositis including its expression at mRNA level in patient muscle tissue, CCL2 protein expression in endothelial cells, as well as higher levels of CCL2 in muscle tissue of IIM patients^31,32^. Moreover, we could infer that CCL2 synthesis could be induced by membrane attack complex^24^ formation in endothelial cells surface of DM patients, the main phenotype in our study. Otherwise, there are few reports which suggest the consideration of this cytokine as an early biomarker for ILD development, since CCL2 elevated levels have been observed in DM/PM patients with ILD complication^33,34^.

Once we characterized the damage and muscle strength as well as immunological profile, we looked for correlations between these parameters. We did not find any association according to the MMT8 score, however, one of the most remarkable findings is the association of LDH enzyme with immunological profile, reflected in: the correlation between LDH and IFN-γ serum levels, the difference of IFN-γ serum levels according to normal or elevated LDH levels, higher LDH levels in seropositive patients, as well as a tendency to the higher frequency of seropositive patients with LDH elevated levels.

Concerning a specific histopathological finding and its association with immunological profile, we observed that the most common finding according to HE results, was perivascular inflammatory infiltrate. We also observed a decreased MMT8 score and increased muscle enzymes when there was presence of regeneration markers (basophilic fibers), as well as lower muscle strength and IL-18 elevated serum levels when the patients had endomysial inflammatory infiltrate.

This infiltrate in muscle tissue is commonly composed by CD8^+^ T cells, which could have an association with this cytokine since it has been proved that IL-18 receptor (IL-18R) has higher expression in functional phenotypes of CD8^+^ T cells^35^, meanwhile other studies have demonstrated that exhausted CD8^+^ T cells downregulates its IL-18R expression^36^. In addition, IL-18 has been proposed as a biomarker in IIM due to its correlation with disease activity^37^, and its increment in serum as consequence of muscle damage^38^. Despite the low number of patients in our study, we were able to find an association of higher IL-18 levels in patients with endomysial infiltrate probably due to an active state of immune response mainly orchestrated by CD8^+^ T cells.

Moreover, the most common findings by SEM included myofiber surface irregularities, altered muscle morphology and non-linear muscle fibers. We observed lower levels of IL-1β and CXCL8 in patients with altered muscle morphology, probably due to a chronic damage.

In search of a possible association between HE and SEM findings (Supplementary Table 3), we observed a tendency of basophilic fibers when they were lineal without cellular infiltrate, which could suggest that patients with these features are probably in clinical remission and/or muscle regeneration.

Regarding immunosuppressive treatment, we found lower levels of CXCL8 in patients treated with prednisone. We also found a tendency to diminished levels of IL-23 in patients treated with mofetil mycophenolate, furthermore, we could not detect IL-1β neither IL-17A, important cytokines for IIM immunopathogeny^22^, in the serum of these patients.

Prednisone is a synthetic glucocorticoid biologically inert and converted to prednisolone in the liver, whose immunosuppressant activity is caused by the inhibition of prostaglandin and leukotriene synthesis, molecules implicated in vascular and cellular processes of inflammation which reduces vasodilatation, capillary permeability and the leukocyte migration^39^. Regarding CXCL8, it is a chemokine secreted by endothelial cells in the inflammation site which possesses a role in leukocyte recruitment and transmigration^40^, moreover, if the prednisone reduces the vasodilatation and capillary permeability, we could infer there is no stimulation of the endothelial cells and the CXCL8 serum concentration decreases in these patients.

Concerning mofetil mycophenolate, it is an inhibitor of the inosine-5'-monophosphate dehydrogenase, an enzyme responsible for de novo synthesis of guanine nucleotides, an important process for the proliferation of T and B lymphocytes^40^. This treatment showed the highest quantity of diminished serum levels of cytokines (Supplementary Table 1), probably because of the direct inhibition of immune cell proliferation which is reflected in a lower autoantibody production, as well as lower cytokine and chemokine production. There are no previous reports regarding the direct effect of mycophenolate in IL-23, IL-1β and IL-17A synthesis, however, these three cytokines are involved in an axis that amplifies the proliferation of Th17 cells which produce IL-17A, a cytokine that in turn, stimulates the IL-1, IL-6 and TNF-α production^41^; moreover, if mofetil mycophenolate diminishes the leukocyte proliferation, these are not able to differentiate, consequently the IL-23, IL-1 and IL-17 axis is interrupted, thus, it could explain why IL-23 levels are diminished and IL-17 and IL-1β levels were not detected in our patients under mofetil mycophenolate treatment. It is important to denote that the implication of IL-17 and IL-23 in IIM has been previously reported, observed as an increment of both cytokines in the supernatant of the ex vivo culture of peripheral blood mononuclear cells of IIM patients with a recent disease establishment when compared with patients with established disease^42^. Likewise, it has been reported an increment of IL-23 serum levels in IIM patients as well as a higher expression of the cytokine in impaired muscle tissue, even IL-23 has been proposed as a therapeutic target for IIM treatment^43^.

When we analyzed if there were differences in cytokine and chemokine serum levels depending on the quantity of immunosuppressive drugs prescribed, we observed that IL-18 remained elevated even in patients treated with triple therapy, thus we can highlight the importance of IL-18 in the disease activity as well as the fact that there are cytokines that even under immunosuppressive treatment cannot be diminished^44^.

In summary, we were able to find and describe muscle fiber ultrastructure with marked irregularities, porosities, disruption in the linearity and integrity of the fascicle, more evident in patients with increased serum levels of muscle enzymes and diminished muscle strength. Likewise, there was a negative correlation between the increment of muscle enzymes and the MMT8 score.

We did not observe a particular association of clinical phenotype with histopathological nor serological findings, moreover, is important to consider the clinical stage of the IIIM (most of them in clinical remission). Concerning the association between immunological parameters, the serum levels of IFN-γ are higher in MSA/MAA seropositive patients. Regarding to muscle and immunological parameters association, the LDH enzyme even unspecific of muscle damage showed association to immunological profile.

In conclusion, despite the scarce reports about the use of SEM as a tool in all clinical phenotypes of IIM, our work provides an excellent opportunity to discuss and reframe its clinical usefulness in the diagnostic approach of IIM.

## Methods

### Patients

Twelve patients classified with IIM according to EULAR/ACR 2017 criteria, attending Hospital Civil de Guadalajara “Dr. Juan I. Menchaca” were enrolled in this cross-sectional study. We also included twelve clinically healthy subjects as control group for cytokines and chemokines serum levels comparison. Informed written consent was obtained from every patient and subject before enrollment in the study. This protocol was approved by Ethical and Research committees of Hospital Civil de Guadalajara “Dr. Juan I. Menchaca” (Jalisco state registration number 0318/19 HCJIM/2019).

We obtained peripheral blood and muscle samples of each patient and all our experiments were performed in accordance with relevant guidelines and regulations. Muscle tissue was obtained from the quadriceps by a muscle biopsy procedure performed by a surgeon. Immediately the muscle tissue was stored as corresponding: buffered formaldehyde 10% for HE staining and paraformaldehyde 4% for SEM.

### Manual muscle testing 8 (MMT8) score

The muscle weakness examination was performed by a rheumatologist using the MMT8 score. This tool included seven proximal muscle groups tested bilaterally (deltoids, biceps, wrist extensors, quadriceps, ankle dorsiflexors, gluteus medius, gluteus maximus) and neck flexors which complete the eight muscle groups tested. Each muscle subgroup receives a score of ten points obtaining a maximum score of 150 when the patient did not present muscle weakness^10^.

### Myositis disease activity assessment visual analogue scale (MYOACT)

Disease activity is defined as potentially reversible clinically evident pathology or physiology resulting from the myositis disease process according to the International Myositis Assessment and Clinical Studies Group (IMACS), we evaluated the disease activity in each of our patients using the Disease Activity Core Set Measure, the MYOACT^45^.

### Myositis damage index (MDI)

Damage is defined as persistent changes in anatomy, physiology, pathology or function, which are present for at least 6 months according to the IMACS, we evaluated the damage in each of our patients employing the recommended Disease Damage Core Set Measure, the MDI^45^.

### Hematoxylin–eosin staining

Once the muscle tissues were obtained, they were processed by a pathologist using a validated protocol for hematoxylin–eosin staining. The muscle sample was fixed from 24 to 48 h in 10% neutral buffered formalin. The samples were dehydrated in increasing ethanol solutions, cleared in xylol, and embedded in paraffin on an automatic tissue processor (Leica Biosystems, TP1020). Subsequently, 3 μm sections were obtained and stained with the conventional hematoxylin and eosin technique, and then evaluated by light microscopy (Microscope Carl Zeiss Axio Lab.A1).

Patients were grouped according to histological features in connective tissue increased, diffuse and multifocal atrophy, perifascicular atrophy, perivascular inflammatory infiltrate, endomysial inflammatory infiltrate, degenerated fibers, necrotic fibers, basophilic fibers, edema and bleeding, muscle fiber size variation, endothelial hyperplasia, and tumefaction.

### Scanning electron microscopy

Samples were processed according to validation guidelines by an expert in electronic microscopy. Muscle was fixed with glutaraldehyde 2.5% and phosphate-buffered saline (PBS) 0.1 M, pH 7.2 for twelve hours, subsequently tissue was washed with PBS 0.1 M, pH 7.2 three times. Samples were dehydrated with increasing concentrations of ethanol solutions (30%, 50%, 70%, 90%, 100%, 100%), each step had a duration of fifteen minutes. Posteriorly they were collocated in hexamethyldisilazane with absolute alcohol (1:1) for fifteen minutes, then tissues were reposed in hexamethyldisilazane for fifteen minutes and subsequently were dried with the air flux of the extraction hood (Burdinola, BST1200). The dehydrated tissues were prepared in slides with carbon tape and were then coated with a layer of gold by a sputter coater (Denton vacuum, Desk V). Finally, the samples were observed in the scanning electronic microscope (JEOL, JSM6610LV).

Patients were grouped according to ultrastructural findings in non-linear muscle fibers, myofiber surface irregularities, altered muscle morphology, muscle fibers perforation, cellular infiltrates, size fiber variation, and myofiber surface porosities.

### Autoantibodies detection

Autoantibodies were detected in serum samples of our patients using the line-blot assay kit Euroline: Autoimmune Inflammatory Myopathies from EUROIMMUN Medizinische Labordiagnostika AG (31 Seekamp. Lübeck, DE 23,560) according to test instructions. This kit allows the detection of twelve MSA (anti- Mi-2α, Mi-2β, TIF1γ, MDA5, NXP2, SAE1, Jo-1, SRP, PL-7, PL-12, EJ, OJ) and four MAA (Ku, PM-Scl100, PM-Scl75, Ro-52). This kit includes test strips coated with highly purified and biochemically characterized autoantigens which were incubated with diluted serum samples and, in the case of positivity, the autoantibodies bounded to the corresponding site and catalyzed a color reaction. For the correct evaluation of the test strips, they were read by EUROLineScan software and the results were interpreted according to the signal intensity of the bands.

### Cytokines and chemokines quantification

Serum levels of cytokines (IL-1β, IFN-α2, IFN-γ, TNF-α, IL-6, IL-10, IL-12p70, IL-17A, IL-18, IL-23, IL-33) and chemokines (CCL2, CXCL8) were quantified using the kit LEGENDplex Multi-Analyte Flow Assay Kit from BioLegend (9727 Pacific Heights Blvd. San Diego, CA 92,121) following test instructions as corresponding. This multiple bead-based immunoassay is based on the basic principle of ELISA type sandwich assay. Each bead is coated with a specific antibody for the cytokine or chemokine of interest, subsequently, these beads were differentiated by size and internal fluorescence intensity using a flow cytometer (Applied Biosystems, Attune NxT). Measures were analyzed in the online software of LEGENDplex (https://legendplex.qognit.com/).

### Complementary laboratory data

ESR and CRP as inflammatory mediators were measured using Wintrobe’s method and nephelometry, respectively. Serum levels of muscle enzymes (CPK, AST, ALT, LDH and aldolase) were considered as muscle damage marker, they were recorded at the time of diagnosis and recruitment.

### Settings and equipment

The microscope employed to performed Light Microscopy (Leica Mycrosystems, DM500 RH) features 10x (PLAN 10x/0.22 OFN 20, Leica) and 40 × objectives (PLAN 40x/0.65 OFN 20, Leica) as well as an ocular HC PLAN s 10x/20. For image acquisition we utilized the microscope camera (ICC50 W, serial number 4521073915, Leica) and the software Leica Acquire for iMAC, version 3.4.7. Blank adjustment to the pictures was made and pictures were edited in Figma, online version, only for fitting in the panel.

### Statistical analysis

Variables were assessed for normality using the Kolmogorov–Smirnov test; values are presented as mean ± SD, or percentages (%), as appropriate. Comparisons were made using Mann–Whitney U test with Fisher’s exact test for quantitative variables, due the low number of patients, meanwhile qualitative variables were analyzed using χ2 or Fisher’s exact test as appropriate.

Our data were analyzed using SPSS 24.0 software (SPSS Inc. Chicago, IL) and GraphPad Prism version 6.00 for Windows (GraphPad Software, La Jolla, CA), considering a two-tailed level of *P* < 0.05 to be significant for analysis.

## Supplementary Information

Supplementary Information 1.

Supplementary Information 2.

Supplementary Information 3.

## Data Availability

The datasets generated and/or analysed during the current study are available in the FigShare repository. Raw data and original images from microscopes are available in https://doi.org/10.6084/m9.figshare.19593667 and https://doi.org/10.6084/m9.figshare.19830025, respectively.
