# Supplementary material for: Impaired muscle strength is associated with ultrastructure damage in myositis
Source: Sci Rep. 2022 Oct 21;12:17671. doi: 10.1038/s41598-022-22754-4 (PMC9586957; doi:10.1038/s41598-022-22754-4)
Supplement: Supplementary file 1 — Supplementary Information 1. [file 41598_2022_22754_MOESM1_ESM.docx]

**Supplementary Table 1.** Serum levels of cytokines and chemokines according to seropositivity.

| **Serum levels (pg/mL)** | **Seropositive patients (n = 5)** | **Detection rate**  **n (%)** | **Seronegative patients**  **(n = 7)** | **Detection rate**  **n (%)** | ***P*^#^*** |
| --- | --- | --- | --- | --- | --- |
| **IL-1β (**$\bar{\mathbf{x}}\boldsymbol{\pm D.E.}$**)** | 5.8 ± 8.29 | 2 (40.0) | 1.2 ± 1.57 | 3 (42.9) | 0.691 |
| **IFN-α2 (**$\bar{\mathbf{x}}\boldsymbol{\pm D.E.}$**)** | 2.6 ± 1.76 | 5 (100.0) | 1.7 ± 1.10 | 7 (100.0) | 0.343 |
| **IFN-γ (**$\bar{\mathbf{x}}\boldsymbol{\pm D.E.}$**)** | 12.1 ± 10.80 | 4 (80.0) | 1.7 ± 2.23 | 3 (42.9) | **0.048** |
| **TNF-α (**$\bar{\mathbf{x}}\boldsymbol{\pm D.E.}$**)** | 0.0 | 0 | 0.0 | 0 | --- |
| **IL-6 (**$\bar{\mathbf{x}}\boldsymbol{\pm D.E.}$**)** | 14.5 ± 9.80 | 5 (100.0) | 9.0 ± 5.48 | 6 (85.7) | 0.639 |
| **IL-10 (**$\bar{\mathbf{x}}\boldsymbol{\pm D.E.}$**)** | 1.2 ± 0.81 | 5 (100.0) | 1.2 ± 1.58 | 6 (85.7) | 0.639 |
| **IL-12p70 (**$\bar{\mathbf{x}}\boldsymbol{\pm D.E.}$**)** | 0.0 | 0 | 0.0 | 0 | --- |
| **IL-17A (**$\bar{\mathbf{x}}\boldsymbol{\pm D.E.}$**)** | 0.1 ± 0.29 | 1 (20) | 0.2 ± 0.41 | 2 (28.6) | 0.876 |
| **IL-18 (**$\bar{\mathbf{x}}\boldsymbol{\pm D.E.}$**)** | 258.0 ± 150.71 | 5 (100.0) | 473.9 ± 880.47 | 6 (85.7) | 0.639 |
| **IL-23 (**$\bar{\mathbf{x}}\boldsymbol{\pm D.E.}$**)** | 3.1 ± 2.94 | 5 (100.0) | 43.1 ± 105.55 | 7 (100.0) | 0.432 |
| **IL-33 (**$\bar{\mathbf{x}}\boldsymbol{\pm D.E.}$**)** | 6.8 ± 10.20 | 2 (40.0) | 1.6 ± 4.27 | 1 (14.3) | 0.432 |
| **CCL2 (**$\bar{\mathbf{x}}\boldsymbol{\pm D.E.}$**)** | 294.8 ± 85.11 | 5 (100.0) | 116.3 ± 66.41 | 7 (100.0) | **0.005** |
| **CXCL8 (**$\bar{\mathbf{x}}\boldsymbol{\pm D.E.}$**)** | 3.3 ± 2.96 | 4 (80.0) | 3.0 ± 5.02 | 4 (57.1) | 0.530 |

**(**$\bar{\mathbf{x}}$**):** mean; **S.D.:** Standard Deviation; **pg:** picogram; **mL:** milliliter; **IL:** interleukin; **IFN:** interferon; **TNF:** Necrosis Tumor Factor; **CCL2:** chemokine (C-C motif) ligand 2; **CXCL8:** chemokine (C-X-C motif) ligand 8. *We compared cytokine and chemokine serum levels between seropositive and seronegative patients. ^#^Mann-Whitney U test with Fisher’s exact test.
