# Supplementary material for: Impaired muscle strength is associated with ultrastructure damage in myositis
Source: Sci Rep. 2022 Oct 21;12:17671. doi: 10.1038/s41598-022-22754-4 (PMC9586957; doi:10.1038/s41598-022-22754-4)
Supplement: Supplementary file 2 — Supplementary Information 2. [file 41598_2022_22754_MOESM2_ESM.docx]

**Supplementary Table 2.** Serum levels of cytokines and chemokines according to immunosuppressive treatment.

| **Serum levels (pg/mL)** | **Prednisone** | | | **Hydroxychloroquine** | | | **Mycophenolate** | | |
| --- | --- | --- | --- | --- | --- | --- | --- | --- | --- |
|  | **Yes**  **(n = 8)** | **No**  **(n = 3)** | ***P*^#^** | **Yes**  **(n = 4)** | **No**  **(n = 7)** | ***P*^#^** | **Yes**  **(n = 3)** | **No**  **(n = 9)** | ***P*^#^** |
| **IL-1β (**$\bar{\mathbf{x}}\boldsymbol{\pm S.D.}$**)** | 4.0 ± 6.80 | 1.7 ± 1.77 | 0.782 | 7.2 ± 8.81 | 1.2 ± 1.57 | 0.452 | 0.0 | 4.7 ± 6.48 | 0.170 |
| **IFN-α2 (**$\bar{\mathbf{x}}\boldsymbol{\pm S. D.}$**)** | 2.3 ± 1.64 | 1.8 ± 0.78 | 0.776 | 2.6 ± 1.17 | 1.9 ± 0.95 | 0.927 | 2.3 ± 1.17 | 2.1 ± 1.59 | 0.776 |
| **IFN-γ (**$\bar{\mathbf{x}}\boldsymbol{\pm S.D.}$**)** | 7.5 ± 10.31 | 3.2 ± 2.81 | > 0.999 | 9.1 ± 12.60 | 4.8 ± 6.77 | 0.712 | 9.5 ± 8.37 | 5.2 ± 9.41 | 0.255 |
| **TNF-α (**$\bar{\mathbf{x}}\boldsymbol{\pm S.D.}$**)** | 0.0 | 0.0 | --- | 0.0 | 0.0 | --- | 0.0 | 0.0 | --- |
| **IL-6 (**$\bar{\mathbf{x}}\boldsymbol{\pm S.D.}$**)** | 12.6 ± 8.49 | 7.5 ± 6.70 | 0.497 | 13.9 ± 7.19 | 9.6 ± 8.63 | 0.527 | 13.6 ± 10.90 | 10.3 ± 7.43 | 0.630 |
| **IL-10 (**$\bar{\mathbf{x}}\boldsymbol{\pm S.D.}$**)** | 1.4 ± 1.53 | 0.8 ± 0.36 | 0.952 | 1.1 ± 0.92 | 1.3 ± 1.56 | 0.824 | 2.1 ± 2.22 | 0.9 ± 0.75 | 0.297 |
| **IL-12p70 (**$\bar{\mathbf{x}}\boldsymbol{\pm S.D.}$**)** | 0.0 | 0.0 | --- | 0.0 | 0.0 | --- | 0.0 | 0.0 | --- |
| **IL-17A (**$\bar{\mathbf{x}}\boldsymbol{\pm S.D.}$**)** | 0.1 ± 0.23 | 0.5 ± 0.52 | 0.103 | 0.2 ± 0.32 | 0.2 ± 0.41 | > 0.999 | 0.0 | 0.3 ± 0.41 | 0.491 |
| **IL-18 (**$\bar{\mathbf{x}}\boldsymbol{\pm S.D.}$**)** | 518.1 ± 796.37 | 97.3 ± 37.66 | 0.133 | 809.9 ± 1104.53 | 171.0 ± 146.84 | 0.164 | 261.2 ± 180.40 | 456.6 ± 817.67 | 0.921 |
| **IL-23 (**$\bar{\mathbf{x}}\boldsymbol{\pm S.D.}$**)** | 38.5 ± 98.59 | 2.8 ± 1.97 | 0.497 | 5.3 ± 2.99 | 42.2 ± 105.93 | 0.412 | 1.2 ± 0.48 | 39.1 ± 98.33 | 0.085 |
| **IL-33 (**$\bar{\mathbf{x}}\boldsymbol{\pm S.D.}$**)** | 2.9 ± 8.07 | 7.5 ± 6.52 | 0.152 | 5.7 ± 11.41 | 3.2 ± 5.51 | > 0.999 | 3.8 ± 6.51 | 4.3 ± 8.48 | > 0.999 |
| **CCL2 (**$\bar{\mathbf{x}}\boldsymbol{\pm S.D.}$**)** | 203.1 ± 120.67 | 196.8 ± 124.78 | > 0.999 | 245.0 ± 157.78 | 176.5 ± 88.07 | 0.527 | 228.3 ± 82.65 | 191.3 ± 129.00 | 0.497 |
| **CXCL8 (**$\bar{\mathbf{x}}\boldsymbol{\pm S.D.}$**)** | 1.6 ± 1.50 | 8.3 ± 5.59 | **0.018** | 1.6 ± 1.99 | 4.4 ± 4.91 | 0.412 | 4.2 ± 3.25 | 3.1 ± 4.67 | 0.485 |

$\bar{\mathbf{x}}$**:** mean; **S.D.**: standard deviation; **pg:** picogram; **mL:** milliliter; **IL:** interleukin; **IFN:** interferon; **TNF:** Necrosis Tumor Factor; **CCL2:** chemokine (C-C motif) ligand 2; **CXCL8:** chemokine (C-X-C motif) ligand 8. ^#^Mann-Whitney U test with Fisher’s exact test.
