# Supplementary material for: Impaired muscle strength is associated with ultrastructure damage in myositis
Source: Sci Rep. 2022 Oct 21;12:17671. doi: 10.1038/s41598-022-22754-4 (PMC9586957; doi:10.1038/s41598-022-22754-4)
Supplement: Supplementary file 3 — Supplementary Information 3. [file 41598_2022_22754_MOESM3_ESM.docx]

**Supplementary Table 3.** Association of muscle histopathological findings by hematoxylin-eosin and scanning electron microscopy techniques.

| **Hematoxylin-eosin findings** | | **Scanning electron microscopy findings** | | | | | | | | | | | |
| --- | --- | --- | --- | --- | --- | --- | --- | --- | --- | --- | --- | --- | --- |
|  |  | **Altered muscular morphology** | | | **Non-linear muscular fibers** | | | **Cellular infiltrate** | | | **Myofiber surface porosities** | | |
|  |  | **Yes** | **No** | ***P*^#^** | **Yes** | **No** | ***P*^#^** | **Yes** | **No** | ***P*^#^** | **Yes** | **No** | ***P*^#^** |
| **Perivascular inflammatory infiltrate** | **Yes** | 3 | 2 | > 0.999 | 3 | 2 | > 0.999 | 2 | 3 | > 0.999 | 1 | 2 | > 0.999 |
|  | **No** | 3 | 2 |  | 3 | 1 |  | 3 | 2 |  | 2 | 2 |  |
| **Perifascicular atrophy** | **Yes** | 3 | 1 | 0.571 | 2 | 1 | > 0.999 | 1 | 3 | 0.524 | 1 | 0 | 0.429 |
|  | **No** | 3 | 3 |  | 4 | 2 |  | 4 | 2 |  | 2 | 4 |  |
| **Endomysial inflammatory infiltrate** | **Yes** | 3 | 1 | 0.571 | 1 | 2 | 0.226 | 1 | 3 | 0.524 | 1 | 1 | > 0.999 |
|  | **No** | 3 | 3 |  | 5 | 1 |  | 4 | 2 |  | 2 | 3 |  |
| **Basophilic fibers** | **Yes** | 2 | 1 | > 0.999 | 0 | 2 | **0.083** | 0 | 3 | 0.167 | 1 | 1 | > 0.999 |
|  | **No** | 4 | 3 |  | 6 | 1 |  | 5 | 2 |  | 2 | 3 |  |
